# Relationship Between Self-Perceived Health, Vitality, and Posttraumatic Growth in Liver Transplant Recipients

**DOI:** 10.3389/fpsyg.2019.01367

**Published:** 2019-06-11

**Authors:** Jesús Funuyet-Salas, Agustín Martín-Rodríguez, Mercedes Borda-Mas, María Luisa Avargues-Navarro, Miguel Ángel Gómez-Bravo, Manuel Romero-Gómez, Rupert Conrad, María Ángeles Pérez-San-Gregorio

**Affiliations:** ^1^Department of Personality, Assessment, and Psychological Treatment, Faculty of Psychology, University of Seville, Seville, Spain; ^2^Hepatic-Biliary-Pancreatic Surgery and Liver Transplant Unit, University Hospital Virgen del Rocío, Seville, Spain; ^3^Digestive Diseases Unit, University Hospital Virgen del Rocío, Seville, Spain; ^4^Department of Psychosomatic Medicine and Psychotherapy, University of Bonn, Bonn, Germany

**Keywords:** liver transplantation, posttraumatic growth, self-perceived health, vitality, patients

## Abstract

Our objective was to analyze the differences in posttraumatic growth in 240 liver transplant recipients based on two factors. First, self-perceived health: better (Group 1 = G_1_) and worse (Group 2 = G_2_). Second, vitality: more (Group 3 = G_3_) and less (Group 4 = G_4_). The Posttraumatic Growth Inventory, SF-36 Health Survey (Item 2) and SF-12 Health Survey (vitality dimension) were used. Firstly, analyzing main effects recipients with better (G_1_) compared to worse (G_2_) self-perceived health, showed greater posttraumatic growth. Interaction effects were found on essential posttraumatic growth domains such as new possibilities (*p* = 0.040), personal strength (*p* = 0.027), and appreciation of life (*p* = 0.014). Statistically significant differences showed that among transplant recipients with worse self-perceived health (G_2_), those with more vitality had higher levels on abovementioned posttraumatic growth dimensions. However, in transplant recipients with better self-perceived health (G_1_) respective dimensions were not significantly influenced by the level of vitality. Among the recipients with less vitality (G_4_), those with better self-perceived health showed higher scores on abovementioned posttraumatic growth dimensions. We conclude that positive self-perceived health might compensate for a lack of vitality as well as a high level of vitality may compensate for negative self-perceived health regarding the development of crucial aspects of posttraumatic growth after liver transplantation.

## Introduction

At the time of insufficiency or failure of a vital organ, transplantation presents an effective therapeutic alternative offering longer and better quality of life (Kugler et al., 2013). Specifically, liver transplantation is the best option when acute liver disease is triggered with severe short-term prognosis (Karam et al., 2003; Sirivatanauksorn et al., 2012).

Liver transplantation is a critical and determinant moment in life. In general, it gives hope of reestablishing a severely harmed state of health and quality of life, frequently after having lived with the disease and dysfunctionality for a long time (Ziȩba et al., 2015). However, transplantation may be a traumatic and highly stressful experience, among other reasons, because of the risks involved. Among these are death, relapse of the disease and dependency on immunosuppressants which may negatively interfere with the recipient’s quality of life (Grinyó et al., 2012; Pérez-San-Gregorio et al., 2012). Fantasies about the donor, symptoms of anxiety, depression and posttraumatic stress, or rejection of body image are psychological problems that usually appear after transplantation (Pérez-San-Gregorio et al., 2005; Baranyi et al., 2013; Annema et al., 2015).

The birth of positive psychology in the 1990s motivated development of a salutogenic perspective promoting health by studying, for example, strengths of individuals after a traumatic experience (Wu et al., 2015; Martz and Livneh, 2016). From this perspective, the focus of attention ceases to be placed exclusively on problems derived from transplantation by concentrating on the possibility of developing a positive transformation of life attributed to this traumatic experience (Anand-Kumar et al., 2014). Thus emerged the concept of posttraumatic growth, which alludes to a subjective experience of positive psychological change as a consequence of living through a highly stressful situation (Tedeschi and Calhoun, 2004), which challenges a person’s most basic core beliefs, self-concept and setting. It also favors elaboration of new cognitive schemas and development of different coping strategies (Tedeschi and Calhoun, 1995; Martins-da-Silva et al., 2011).

Posttraumatic growth has been widely studied in cancer patients (Casellas-Grau et al., 2018; Sharp et al., 2018; Tobin et al., 2018) and in those who have undergone hematopoietic stem cell transplantation (Forinder and Norberg, 2014; Jeon et al., 2015; Rosenberg et al., 2015). However, there has been relatively few research in liver transplant recipients. On the one hand, respective studies indicate that posttraumatic growth increases identification of recipients with their family and with other recipients (Scrignaro et al., 2016). On the other hand, they point to a close association between development of strong posttraumatic growth and the use of an affective, predominantly positive tone in telling about past life events (Ziȩba et al., 2015). Pérez-San-Gregorio et al. (2017b) also showed that a high level of posttraumatic growth is related to more use of adaptive, healthy coping strategies.

Other studies on posttraumatic growth and quality of life did not find a significant positive relationship between these two factors, such as the one by Moore et al. (2011) with a sample of 202 patients diagnosed with hepatobiliary carcinoma. A similar conclusion was found in a study by Fox et al. (2014) with 64 lung transplant recipients, which found only a minimal association between posttraumatic growth and quality of life related to physical functioning.

However, to date it is still unclear which mechanisms underlie the development of posttraumatic growth (Tedeschi and Calhoun, 2004). Nevertheless, it is clear that it involves cognitive and affective-motivational processes to be able to restructure cognitive schemata and their emotional underpinnings. In the context of posttraumatic growth after liver transplantation the construct of self-perceived health is very relevant. There is growing evidence for its importance regarding quality of life across a wide spectrum of disease entities. Thus, its influence on quality of life has been demonstrated in patients with cancer (Cameron et al., 2012; Hirsch et al., 2012), cardiovascular pathology (Bachmann et al., 2016; Ko and Boo, 2016), hepatitis and HIV (Marcellin et al., 2011; Elliott et al., 2017; Zhu et al., 2017). A study by Martín-Rodríguez et al. (2012) demonstrated the influence of self-perceived health on mental health in cirrhosis patients on the transplant waiting list and liver transplant recipients. According to a study by Pérez-San-Gregorio et al. (2013) on 168 liver transplant recipients, those with worse self-perceived health showed worse quality of life than those with better self-perceived health, especially in the bodily pain and general health dimensions. Against this backdrop, self-perceived health can be seen as a construct which can assist in predicting a patient’s affective development and potential posttraumatic growth after liver transplantation.

A second construct closely linked to posttraumatic growth is vitality (Tedeschi and Calhoun, 1996, 2004). A “positive feeling of having energy available to the self” (Nix et al., 1999, p. 266) is a widely accepted definition, accentuating the aspect of subjectively assessing one’s own emotional state. Even though self-perceived health and vitality are regarded as closely associated as, for example, in the construction of the SF-36, there is some evidence (Guérin, 2012) that it makes sense to disentangle them, as self-perceived health embraces the cognitive component of health-related self-assessment, whereas (self-perceived) vitality its affective-motivational component.

Against this backdrop, our study analyzes the differences in posttraumatic growth after liver transplantation as a function of two factors, self-perceived health and vitality. We specifically hypothesized that better self-perceived health and higher vitality of transplant recipients may mutually facilitate higher posttraumatic growth.

## Materials and Methods

### Participants

This research was approved by the Ethics Committee of the Virgen del Rocío University Hospital of Seville. At the beginning of recruitment all 569 patients still alive from a total clinical sample of 1053 recipients who had undergone transplantation surgery at the Virgen del Rocío University Hospital in Seville from 1990 to 2014 were informed about the possibility of study participation by the Association of Liver Transplant Recipients and the Hepatic-Biliary-Pancreatic Surgery and Liver Transplant Unit. Inclusion criteria for participants were as follows: (a) over 18 years of age, (b) informed consent, (c) reception of only one liver transplant. Exclusion criteria were (a) difficulties in understanding the evaluation instruments, (b) severe or disabling psychiatric disorder. The recruited sample consisted of 240 patients, 185 men and 55 women, with a mean age of 60.21 (*SD* = 9.30) years. Of the recipients, 61.7, 22.5, and 15.8% had a low (did not complete high school), intermediate (high school education), and higher formal education (A level), respectively. For further details, see Pérez-San-Gregorio et al. (2017a).

### Instruments

Each participant filled out the 21 items on the *Posttraumatic Growth Inventory* (Tedeschi and Calhoun, 1996) which evaluates perception of personal benefits after experiencing a traumatic event. This instrument is structured in a Likert-type scale from 0 (“I did not experience this change as a result of my crisis”) to 5 (“I experienced this change to a very great degree as a result of my crisis”) in the positive direction.

The scale includes five domains of posttraumatic growth named new possibilities, relating to others, personal strength, spiritual change, and appreciation of life. In the Spanish version of this instrument (Weiss and Berger, 2006), we found the following Cronbach’s alphas in our sample of patients: 0.94 for personal strength, 0.88 in relating to others, 0.80 in new possibilities, 0.77 in personal strength, 0.76 in appreciation of life and 0.73 in spiritual change.

To form the various levels of independent variables, the participants answered Item 2 on the Spanish version of the *SF-36 Health Survey* (Alonso et al., 1995) and the vitality subscale of the *12-Item Short-Form Health Survey (SF-12v.2)* (Ware et al., 2002; Maruish, 2012).

### Procedure

A 2 × 2 factorial design was carried out with the independent variables self-perceived health and vitality.

(a) *Self-perceived health*, with two levels (*better* or *worse*). This variable was selected based on the scores on Item 2 of the SF-36 (“Compared to 1 year ago, how would you rate your health in general now?”): (1) G_1_: liver transplant recipients with *better* self-perceived health: patients with scores over 54.2%, which referred to the following answers: “somewhat better now than 1 year ago” and “much better now than 1 year ago,” forming a subgroup of 110 patients, and (2) G_2_: liver transplant recipients with *worse* self-perceived health: patients with scores equal to or less than 54.2%, which referred to the following answers: “about the same than 1 year ago,” “somewhat worse now than 1 year ago” and “much worse now than 1 year ago,” forming a subgroup of 130 patients.

(b) *Vitality*, with two levels (*more* and *less*). This variable was selected based on the scores on the SF-12 vitality dimension (“How much of the time during the past 4 weeks, did you have a lot of energy?”): (1) G_3_: liver transplant recipients with *more* vitality: patients with scores over 45.4%, which referred the following answers: “most of the time” and “all of the time,” forming a subgroup of 131 patients, and (2) G_4_: liver transplant recipients with *less* vitality: patients with scores equal to or less than 45.4%, which referred to the following answers: “some of the time,” “a little of the time” and “none of the time,” forming a subgroup of 109 patients.

To establish the two subgroups corresponding to the factors self-perceived health and vitality, we proceeded as follows: First, the scores of each patient were taken into account for both variables, which varied from 0 to 100. Second, for both variables the scores were ordered from least to most. Afterward the accumulated percentages of the frequency distribution were taken into account two form two subgroups of patients for each variable, which embraced approximately half of the sample. From a clinical perspective, these divisions into two subgroups in each of the factors are very relevant, since they allow the categorization of patients with similar characteristics.

### Statistical Analysis

Pearson’s chi-squared was used to compare the categorical variables (gender, marital status, education, and employment), and for the quantitative variables (age and months since transplantation), the *t*-test for independent samples was applied.

We also applied a covariance analysis to analyze the influence of two independent factors on the level of posttraumatic growth: level of self-perceived health (better or worse) and vitality (more or less). In this analysis, first age of the transplant patient was included as a covariate. In a second analysis age and time since transplantation were included as covariates. Results with *p* < 0.05 were regarded as significant, results with *p* < 0.10 ≥ 0.05 as statistical trend. Effect sizes were calculated using Cohen’s w (for categorical variables) and Cohen’s *d* (for quantitative variables). The data were analyzed with the SPSS 22 statistical program.

## Results

The group of liver transplant recipients with better self-perceived health (G_1_) was made up of 89 men and 21 women with a mean age of 59.38 years (*SD* = 7.68), while the one with worse self-perceived health (G_2_) was made up of 96 men and 34 women, with a mean age of 60.91 (*SD* = 10.46). The group of liver transplant recipients with more vitality (G_3_) was made up of 105 men and 26 women with a mean age of 60.12 (*SD* = 8.79), and the one with less vitality (G_4_) had 80 men and 29 women with a mean age of 60.31 (*SD* = 9.92). The sociodemographic and clinical data for the four groups of liver transplant recipients are summarized in Tables 1, 2.

**Table 1:** Comparison of sociodemographic and clinical variables between two groups with better (G_1_) and worse (G_2_) self-perceived health.

	Level of self-perceived health		Intergroup comparisons	Effect sizes
	Better (G_1_) *n* = 110	Worse (G_2_) *n* = 130		
	*M* (*SD*)	*M* (*SD*)	*t* (*p*)	Cohen’s *d*
Age	59.38	60.91	*t*_(1,233.52)_ = 1.300	−0.166 N
	(7.68)	(10.46)	(0.195)	
Months since	57.17	113.66	*t*_(1,237.96)_ = 7.361	−0.947 L
transplantation	(54.83)	(64.05)	(<0.001)	

	**%**	**%**	**χ^2^ (*p*)**	**Cohen’s *w***

Gender			1.683 (0.195)	−0.084 N
Male	80.9	73.8		
Female	19.1	26.2		
Marital status			3.776 (0.052)	−0.125 S
With partner	73.6	83.8		
Without partner	26.4	16.2		
Education			2.573 (0.276)	0.104 S
Low	63.6	60		
Medium	24.5	20.8		
High	11.9	19.2		
Employment			1.689 (0.194)	0.084 N
Working	5.5	10		
Not working	94.5	90		

*N, null effect size; S, small effect size; L, large effect size.*

**Table 2:** Comparison of sociodemographic and clinical variables between two groups with more (G_3_) and less (G_4_) vitality.

	Level of vitality		Intergroup comparisons	Effect sizes
	More (G_3_) *n* = 131	Less (G_4_) *n* = 109		
	*M* (*SD*)	*M* (*SD*)	*t* (*p*)	Cohen’s *d*
Age	60.12	60.31	*t*_(1,217.91)_ = 0.155	−0.020 N
	(8.79)	(9.92)	(0.877)	
Months since	86.47	89.33	*t*_(1,238)_ = 0.332	−0.042 N
transplantation	(64.20)	(68.77)	(0.740)	

	**%**	**%**	**χ^2^ (*p*)**	**Cohen’s *w***

Gender			1.538 (0.215)	−0.08 N
Male	80.2	73.4		
Female	19.8	26.6		
Marital status			0.009 (0.926)	0.006 N
With partner	79.4	78.9		
Without partner	20.6	21.1		
Education			6.737 (0.034)	0.168 S
Low	55	69.7		
Medium	24.4	20.2		
High	20.6	10.1		
Employment			3.037 (0.081)	−0.112 S
Working	10.7	4.6		
Not working	89.3	95.4		

*N, null effect size; S, small effect size.*

With regard to the analysis of socio-demographic variables in better versus worse self-perceived health, there was a statistical trend in the direction of worse self-perceived health in recipients with a partnership (small effect size). Regarding the comparison of more versus less vitality there was a significant difference showing less vitality in recipients having a lower level of education with a small effect size. Furthermore, those recipients not working showed a statistical trend toward less vitality (small effect).

Regarding clinical variables those recipients with longer time since transplantation showed significantly poorer self-perceived health with a large effect size (*p* < 0.001, *d* = −0.947; Table 1).

**FIGURE 1:**
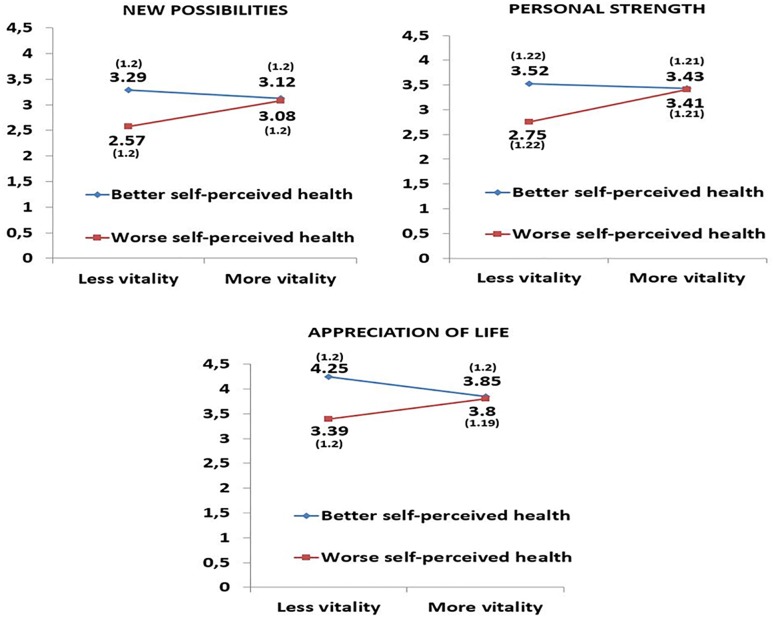
Interaction effects between the two factors self-perceived health (better and worse) and vitality (more and less). Means (standard deviations) adjusted for age of liver transplant recipients.

In the next step of analysis we were interested in differences in posttraumatic growth in above mentioned subgroups controlling for age (Table 3). Regarding the level of posttraumatic growth, interaction effects were found between self-perceived health and vitality factors in the following variables: new possibilities [*F*_(1,233)_ = 4.278, *p* = 0.040], personal strength [*F*_(1,233)_ = 4.951, *p* = 0.027], and appreciation of life [*F*_(1,233)_ = 6.109, *p* = 0.014] (Table 3). Regarding simple effects, as shown in Figure 1 and in Tables 4, 5, we found that among transplant recipients with worse self-perceived health, those with more vitality scored higher on the domains new possibilities (*p* = 0.017, *d* = 0.427), personal strength (*p* = 0.003, *d* = 0.541), and new appreciation of life (*p* = 0.053, *d* = 0.346) the latter by a statistical trend, while those with better self-perceived health showed no differences in those variables (Table 4 and Figure 1). We also found that among transplant recipients with less vitality, those with better self-perceived health showed higher scores (more posttraumatic growth) than those with worse self-perceived health on the scales new possibilities (*p* = 0.005, *d* = 0.598), personal strength (*p* = 0.003, *d* = 0.627), and new appreciation of life (*p* = 0.001, *d* = 0.718) variables, while those with more vitality did not show these differences as a function of self-perceived health (Table 5 and Figure 1).

**Table 3:** Posttraumatic growth in liver transplant recipients based on level of self-perceived health and level of vitality with age as covariate.

	Level of self-perceived health *M* (*SD*)^1^		Level of vitality *M* (*SD*)^1^		Main effects		Interaction effects
	Better *n* = 110	Worse *n* = 130	More *n* = 131	Less *n* = 109	Self-perceived health *F*_(1,233)_ *p* (*d*^2^)	Vitality *F*_(1,233)_ *p* (*d*^2^)	*F*_(1,233)_ *p*
Relating to others	3.41 (1.30)	3.19 (1.20)	3.38 (1.21)	3.22 (1.29)	1.737	1.026	1.409
					0.189 (0.175 N)	0.312 (0.127 N)	0.236
New possibilities	3.20 (1.32)	2.82 (1.22)	3.10 (1.22)	2.93 (1.30)	5.271	1.103	4.278
					0.023 (0.298 S)	0.295 (0.134 N)	0.040
Personal strength	3.47 (1.33)	3.08 (1.24)	3.42 (1.23)	3.13 (1.32)	5.496	2.958	4.951
					0.020 (0.303 S)	0.087 (0.227 S)	0.027
Appreciation of life	4.05 (1.31)	3.59 (1.22)	3.83 (1.22)	3.82 (1.30)	7.695	0.002	6.109
					0.006 (0.363 S)	0.962 (0.007 N)	0.014
Spiritual change	2.30 (1.79)	2.18 (1.66)	2.29 (1.67)	2.20 (1.78)	0.281	0.150	0.327
					0.597 (0.069 N)	0.699 (0.052 N)	0.568
Total score PTGI	70.68 (24.45)	64.03 (22.70)	69.05 (22.69)	65.67 (24.33)	4.691	1.217	3.794
					0.031 (0.281 S)	0.271 (0.143 N)	0.053

*^1^Means (standard deviation): higher scores show more posttraumatic growth. Recipients’ age has been introduced as covariate in the analysis. ^2^Cohen’s d index: N, null effect size; S, small effect size.*

**Table 4:** Simple effects: comparisons between liver transplant recipients with better (G_1_) and worse (G_2_) self-perceived health at each of the levels of vitality.

Level of vitality	Better self-perceived health (G_1_) *n* = 110		Worse self-perceived health (G_2_) *n* = 130	
	*p*	Cohen’s *d*	*p*	Cohen’s *d*
	New possibilities			
More-less	0.502	−0.139 N	0.017	0.427 S
	Personal strength			
More-less	0.737	−0.070 N	0.003	0.541 M
	New appreciation of life			
More-less	0.112	−0.332 S	0.053	0.346 S

*N, null effect size; S, small effect size; M, medium effect size.*

**Table 5:** Simple effects: comparisons between liver transplant recipients with more (G_3_) and less (G_4_) vitality at each level of self-perceived health.

Level of self-perceived health	More vitality (G_3_) *n* = 131		Less vitality (G_4_) *n* = 109	
	*p*	Cohen’s *d*	*p*	Cohen’s *d*
	New possibilities			
Better-worse	0.859	0.003 N	0.005	0.598 M
	Personal strength			
Better-worse	0.925	0.016 N	0.003	0.627 M
	New appreciation of life			
Better-worse	0.813	0.042 N	0.001	0.718 M

*N, null effect size; M, medium effect size.*

Concerning the main effects, we found statistically significant differences among transplant recipients with better and worse self-perceived health in the new possibilities (*p* = 0.023; *d* = 0.298), personal strength (*p* = 0.020; *d* = 0.303), and appreciation of life (*p* = 0.006; *d* = 0.363) variables, and total posttraumatic growth score (*p* = 0.031; *d* = 0.281). Specifically, those transplant recipients with better self-perceived health showed more posttraumatic growth (Table 3).

**FIGURE 2:**
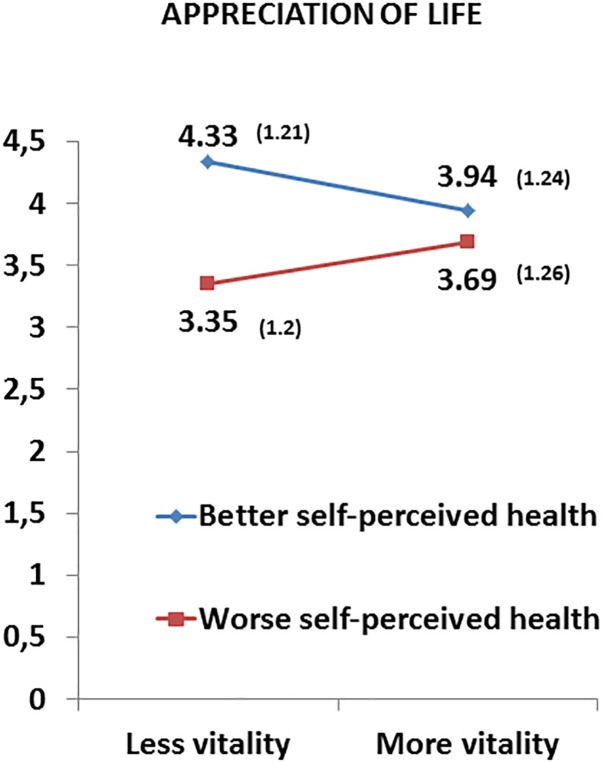
Interaction effects between the two factors self-perceived health (better and worse) and vitality (more and less) in appreciation of life. Means (standard deviations) adjusted for age and time since transplantation of liver transplant recipients.

In a further analysis we looked at the difference in posttraumatic growth controlling for age and time since transplantation (Table 6). A statistically significant interaction effect was found for appreciation of life [*F*_(1,233)_ = 4.799, *p* = 0.029]; the subscales new possibilities [*F*_(1,233)_ = 2.842, *p* = 0.093]; and personal strength [*F*_(1,233)_ = 3.227, *p* = 0.074] showed a statistical trend. Regarding simple effects (Tables 7, 8) we also found that among transplant recipients with less vitality, those with better self-perceived health showed higher scores (more posttraumatic growth) than those with worse self-perceived health on the scale new appreciation of life (*p* < 0.001, *d* = 0.813) as shown in Table 8 and Figure 2.

**Table 6:** Posttraumatic growth in liver transplant recipients based on level of self-perceived health and level of vitality with age and time since transplantation as covariates.

	Level of self-perceived health *M* (*SD*)^1^		Level of vitality *M* (*SD*)^1^		Main effects		Interaction effects
	Better *n* = 110	Worse *n* = 130	More *n* = 131	Less *n* = 109	Self-perceived health *F*_(1,233)_ *p* (*d*^2^)	Vitality *F*_(1,233)_ *p* (*d*^2^)	*F*_(1,233)_ *p*
Relating to others	3.53 (1.34)	3.09 (1.26)	3.37 (1.19)	3.25 (1.27)	6.223	0.539	0.606
					0.013 (0.338 S)	0.464 (0.097 N)	0.437
New possibilities	3.32 (1.35)	2.72 (1.27)	3.08 (1.20)	2.96 (1.29)	11.036	0.617	2.842
					0.001 (0.458 S)	0.433 (0.096 N)	0.093
Personal strength	3.60 (1.37)	2.96 (1.28)	3.40 (1.21)	3.16 (1.30)	12.672	2.047	3.227
					<0.001 (0.483 S)	0.154 (0.191 N)	0.074
Appreciation of life	4.13 (1.18)	3.52 (1.49)	3.82 (1.21)	3.84 (1.30)	11.344	0.020	4.799
					0.001 (0.454 S)	0.886 (-0.016 N)	0.029
Spiritual change	2.44 (1.86)	2.06 (1.74)	2.27 (1.65)	2.23 (1.77)	2.331	0.023	0.054
					0.128 (0.211 S)	0.880 (0.023 N)	0.817
Total score PTGI	73.05 (25.08)	61.76 (23.54)	68.61 (22.27)	66.20 (23.88)	11.606	0.643	2.289
					0.001 (0.464 S)	0.424 (0.104 N)	0.132

*^1^Means (standard deviation): higher scores show more posttraumatic growth. Recipients’ age and time elapsed since transplantation have been introduced as covariates in the analysis. ^2^Cohen’s d index: N, null effect size; S, small effect size.*

**Table 7:** Simple effects on appreciation of life: comparisons between liver transplant recipients with better (G_1_) and worse (G_2_) self-perceived health at each of the levels of vitality.

Level of vitality	Better self-perceived health (G_1_) *n* = 110		Worse self-perceived health (G_2_) *n* = 130	
	*p*	Cohen’s *d*	*p*	Cohen’s *d*
More-less	0.123	−0.318 S	0.118	0.276 S

*S, small effect size.*

**Table 8:** Simple effects on appreciation of life: comparisons between liver transplant recipients with more (G_3_) and less (G_4_) vitality at each level of self-perceived health.

Level of self-perceived health	More vitality (G_3_) *n* = 131		Less vitality (G_4_) *n* = 109	
	*p*	Cohen’s *d*	*p*	Cohen’s *d*
Better-worse	0.293	−0.199 N	<0.001	0.813 L

*N, null effect size; L, large effect size.*

## Discussion

The current study analyses the mutual associations of self-perceived health and vitality on posttraumatic growth in liver transplant recipients. We divided the sample according to better or worse self-perceived health and more or less vitality into four groups. With regard to socio-demographic characteristics in recipients with more versus less vitality there was a statistically significant difference with regard to education. Lower education was associated with a lower level of vitality by a small effect size. Furthermore there was a statistical trend with a small effect size indicating that recipients not working reported less vitality. Comparing recipients with better versus worse self-perceived health those patients having a partnership showed a statistical trend with a small effect size in the direction of worse self-perceived health. As one might have expected in those recipients with worse self-perceived health a significantly longer time-span had passed since transplantation. Particularly long term treatment by immunosuppressants and the associated side effects might have been one important cause for an increase in health problems which corresponds to a decline in self-perceived health (Kugler et al., 2009).

Regarding the influence of self-perceived health and vitality on posttraumatic growth controlling for age significant interaction effects were found on the posttraumatic growth dimensions new possibilities, personal strength and new appreciation of life as opposed to the dimensions relating to others and spiritual change. Further analysis revealed that participants with worse self-perceived health scored significantly higher on abovementioned posttraumatic growth domains when they felt more vitality. On the other hand, in recipients with less vitality, the scores on these dimensions were higher when they had better self-perceived health. When we introduced time since transplantation as covariate in our analysis we found a significant interaction effect on the dimension appreciation of life and the dimensions personal strength and new possibilities showed a statistical trend. Analysis of the simple effects on the dimension appreciation of life revealed similar to the previous analysis that recipients with less vitality scored higher with better self-perceived health.

Previous studies confirm the positive association between self-perceived health and posttraumatic growth. In the article by Fox et al. (2014), lung transplant recipients who experienced more posttraumatic growth showed a better self-perceived general health. Similarly, a meta-analysis of 38 studies of persons diagnosed with cancer or HIV showed evidence that posttraumatic growth was related to better self-perceived physical and mental health (Sawyer et al., 2010). The construct of self-perceived health can be seen as the cognitive component of health-related self-assessment, whereas self-perceived vitality embraces its affective-motivational component. Vitality is characterized by three dimensions (Van Steenbergen et al., 2016): energy, or feeling energized; motivation, that means putting effort in achieving goals; and resilience, which consists of the ability to deal with everyday problems and challenges in life. Thus as our first analysis controlling for age showed having more vitality strengthened posttraumatic growth in those participants who did not realize a satisfactory state of health. Similarly, among the recipients who felt insufficient energy and motivation the awareness of better self-perceived health facilitated the awareness of personal strength, new possibilities and appreciation of life. In this context the close link between cognitive and affective-motivational aspects of mental well-being becomes apparent. Despite a lack of positive thinking the recipient, who feels energized, may realize new opportunities. On the other hand, a lack of energy might be compensated for by positive thoughts of one’s state of health. Respective associations were weaker when controlling for time since transplantation, nevertheless this analysis also revealed a large effect of better self-perceived health on the posttraumatic growth dimension appreciation of life in recipients with less vitality.

This is in line with the theory of posttraumatic growth by Tedeschi and Calhoun (1996, 2004) in which post-traumatic stress is understood to be the engine of post-traumatic growth and cognitive and affective processes are closely intertwined. The degree of posttraumatic growth reported tends to be related to the extent of cognitive engagement or rumination about elements related to the stressful event. The cognitive engagement corresponds to the level of threat associated with the traumatic event. Greater growth has been reported for individuals who reported higher levels of stress or threat (Linley and Joseph, 2004; Weiss, 2004). However, to date it is still not clear why some individuals can grow after a critical event and others are simply overwhelmed by the situation (Tedeschi and Calhoun, 2004). Specific cognitive and affective resources are underlying the ability to grow and according to our findings self-perceived health as well as vitality may be seen as relevant factors in this highly complex process.

In summary, our study could confirm differences in posttraumatic growth of liver transplant recipients according to their self-perceived health and vitality. These results demonstrate potential possibilities for strengthening posttraumatic growth (Jeon et al., 2015). Just as group psychotherapy and cognitive behavioral therapy are performed in cirrhosis patients on the transplantation waiting list (Su et al., 2014; Ramírez et al., 2015), it would be beneficial to implement interventions of this type in the post-transplant stage for the purpose of improving self-perceived health and vitality with potentially beneficial consequences for posttraumatic growth and quality of life. Integrating psychological diagnostics, therapy and outcome evaluation (Geiser et al., 2001) in the protocols for long-term follow-up of liver transplant recipients would facilitate the identification and reduction of psychological risk factors, thereby increasing the likelihood of optimizing recipients’ outcome (Morana, 2009; Dąbrowska-Bender et al., 2018).

Finally, it would be advisable, with a view to future lines of research, to consider some limitations observed in the design of this study. For example, the etiology of the liver disease leading to transplantation was not taken into consideration. There might have been differences in posttraumatic growth between transplantation recipients with alcoholic, viral or metabolic liver cirrhosis. Furthermore, due to its cross-sectional design, it was not possible to analyze the long-term development of specific alterations. A longitudinal study would solve this problem, and could reveal causal relationships between self-perceived health, vitality and posttraumatic growth. Furthermore, there are other variables which could affect the relationship between the above mentioned variables such as personality traits, which were not taken into account.

For future studies a methodological approach based on the narrative theory as suggested by Gangeri et al. (2018) might be interesting to shed light on the complex mechanisms of posttraumatic growth. Thus, instead of quantifying different parameters by questionnaires, it would be important to analyze the personal narrative of recipients about life changes after liver transplantation.

## Ethics Statement

Ethics Committee of the Virgen del Rocío University Hospital of Seville. All subjects gave written informed consent in accordance with the Declaration of Helsinki.

## Author Contributions

All authors conceived and designed the work, revised the manuscript critically for important intellectual content, and approved the final version of the manuscript to be submitted. JF-S, AM-R, RC, and MÁP-S-G performed the bibliography research about the topic, collected, analyzed, and interpreted the data, and drafted the manuscript. MB-M, MLA-N, MÁG-B, and MR-G conceived and designed the work, and analyzed and interpreted the data.

## Conflict of Interest Statement

The authors declare that the research was conducted in the absence of any commercial or financial relationships that could be construed as a potential conflict of interest.
